# A Systematic Calibration Modeling Method for Redundant INS with Multi-Sensors Non-Orthogonal Configuration

**DOI:** 10.3390/mi13101684

**Published:** 2022-10-07

**Authors:** Chunfeng Gao, Guo Wei, Lin Wang, Qi Wang, Zhikun Liao

**Affiliations:** Department of Optoelectronic Engineering, College of Advanced Interdisciplinary Studies, National University of Defense Technology, Changsha 410073, China

**Keywords:** redundant sensors, calibration, inertial measurement unit, ring laser gyroscopes, accelerometers, constant bias, scale factor error, installation error

## Abstract

Because of the non-orthogonal configuration of multi-sensors, the redundant inertial navigation system (INS) has a more complex error model compared with the traditional orthogonal INS, and the complexity of sensors configuration also increases the difficulty of error separation. Based on sufficient analysis of the error principle of redundant IMUs, a generalized high-accuracy calibration modeling method which is suitable for filtering method systematic calibration is summarized in this paper, and it has been applied to an RIMU prototype consisting of four ring laser gyros (RLGs) and four quartz accelerometers. Through the rotational excitation of the three-axis turntable in the laboratory, the high-precision filtering method systematic calibration of the RIMU is achieved, and static navigation and dynamic vehicle test experiments are also carried out. The experimental results reflect that the positioning accuracy can be obviously improved by using this new systematic calibration error model and the validity of this modeling method is also verified.

## 1. Introduction

As the core equipment of inertial navigation systems (INSs), the inertial measurement unit (IMU) is a sensors assembly constructed by accelerometers and gyros. In order to detect faults of the sensors and improve the performance of the system, the redundant IMU (RIMU) has been developed, and it has also been widely used in fighters, satellites, submarines, etc. [1].

IMU calibration is an essential process for the operation of inertial sensors assembled for navigation. The theoretical principle of calibration is to establish an accurate mathematical model of the input and output relationship of an IMU through system identification and parameter estimation [2]. For IMUs, the high-accuracy error calibration is the basis of high precision navigation. There are many kinds of IMU calibration methods which are applied in different situations. As for high-precision IMUs (above tactical level), the commonly used self-calibration methods include analytical calibration and systematic calibration [3].

Analytical calibration requires accurate references given by external equipment (high-precision eight-node hexahedron element or turntable, etc.). Generally, the least square method is used to determine the input and output relationships of gyros and accelerometers on the basis of the IMU calibration model. The accuracy of analytical calibration is limited by the accuracy of the external test equipment, and the effects of shock absorbers deformation cannot be effectively eliminated with shock absorbers (to ensure the stable performance of RLGs, shock absorbers are necessary for the IMUs consisting of mechanically dithered RLGs) [4,5,6].

Systematic calibration uses the IMU navigation errors (position error, velocity error, and attitude error) as the observations to achieve the estimation of the error parameters. Pittman summarizes the advantages of system level calibration as follows: (1) inertial navigation system online self-calibration can be achieved; (2) the calibration accuracy does not depend on the accuracy of the test equipment; and (3) there is no need to record the output of the gyros and accelerometers. The research of Camberlein indicates that the systematic calibration method can make up for the shortcomings of analytical calibration in RLG inertial navigation systems.

There are two ways to conduct the systematic calibration of IMUs: the fitting method and the filtering method. The fitting method is to design a reasonable rotation order to simulate the relationship between the navigation errors and the calibration parameters, where the calibration parameters can be estimated by least square fitting through the navigation errors. The filtering method is to design a high-dimensional Kalman filter to estimate the errors of calibration parameters by using the parameter errors as the filter states and the navigation errors as the observed quantity. Calibration has been cast as a state estimation problem in the Kalman filtering systematic calibration. The error modeling of the Kalman filter is the key technology of filtering systematic calibration. The accuracy and simplicity of the error model directly restrict the accuracy and the convergence speed of calibration [7,8,9,10,11]. 

The calibration of orthogonal IMUs has been carried out since the 1970s. However, the research on the efficient calibration techniques of the non-orthogonal RIMU is still insufficient. Considering the increase of inertial devices and the non-orthogonal installation of sensors, the calibration modeling and errors separation become more difficult [12,13,14]. 

For orthogonal IMUs, the angle transformation of the system is considered as orthogonal transformation, and the attitude matrix is a third-order full rank matrix. Therefore, the sensors installation error of orthogonal IMUs can be considered as small angle errors. For non-orthogonal RIMUs, the angular increment and velocity increment are required to be transformed into orthogonal systems with a non-full rank n×3−order matrix. Thus, the installation errors of the non-orthogonal RIMU should be regarded as coupled errors rather than small angle errors, which makes the error separation process more difficult. Moreover, when the number of inertial sensors increases, the filter order will also increase through filtering systematic calibration and the observability of each parameter will be reduced while the filtering results are more difficult to converge. In this paper, the modeling method of filtering systematic calibration of an RLG redundant inertial navigation system is studied [15,16,17,18].

In order to find a high accuracy calibration method of the non-orthogonal RIMU, the specific main works of the paper are as follows:

- The error model of analytic calibration of a non-orthogonal RIMU is analyzed;
- How to optimize the systematic calibration model is studied;
- The method of systematic calibration based on the filtering method is introduced;
- The accuracy of the model is verified by using an RIMU with four RLGs.

This paper is organized into five sections. In Section 2, the calibration parameters of the non-orthogonal RINS and the error model of analytic calibration are introduced. In Section 3, the systematic calibration model of the non-orthogonal RINS is studied. In Section 4, based on the prototype developed by our research group, the performance of the calibration model is verified. Finally, conclusions are drawn in the last section.

## 2. The Analytical Calibration Parameters and the Error Model of the Non-orthogonal RIMU

The coordinate frames and the angular orientation between them are shown as follows.

(1) Local navigation frame (n): The $z_{n}$ axis is upward and parallel to the local vertical, the $x_{n}$ and $y_{n}$ axes are parallel to the local tangent plan, and the $y_{n}$ axis lies along the northerly direction.

(2) INS body frame (b): The b frame is parallel to the input axes of the IMU with its $x_{b}$ axis parallel to the right side of the IMU and the $y_{b}$ axis is parallel to the heading direction.

(3) Sub body frame (s): The s frame is not always an orthogonal right-handed coordinate frame, and the $i_{s}\;$ axis is parallel to the sensitive axis of the i-th gyro. The angle between the b frame and s frame is fixed, and we use configuration matrix H to represent this angle. 

(4) INS pseudo body frame ($\tilde{b}$): The $\tilde{b}$ frame is a coordinate system converted from the theoretical value of the configuration matrix of the s frame. There are small angle errors in the pseudo $\tilde{b}$ frame and the real b frame. 

Firstly, the error characteristics of the non-orthogonal RINS are analyzed. The redundancy (which represents the number of gyros as well as accelerometers) of the RINS is assumed to be n, and the calibration parameters of the RIMU mainly consist of the following three parts:

1. Constant Bias

The constant bias of a gyro is the output pulse of the gyro when the input angular velocity is zero. Generally, the unit of output pulse is expressed as angular velocity in °/h. The constant bias of an accelerometer is the acceleration output of the accelerometer when the specific force input is zero in μg or mg. The constant bias of gyros (ε) and constant bias of accelerometers ($\vec{\nabla }$) are shown as follows:

(1) $$\varepsilon ={\left[\begin{array}{cccc} \varepsilon_{1} & \varepsilon_{2} & \cdots & \varepsilon_{n} \end{array}\right]}^{T}$$

($\varepsilon_{i}$ is the constant bias of the i-th gyro)

(2) $$\vec{\nabla }={\left[\begin{array}{cccc} \nabla_{1} & \nabla_{2} & \cdots & \nabla_{n} \end{array}\right]}^{T}$$

($\nabla_{i}$ is the constant bias of the i-th accelerometer)

2. Scale Factor Error

The output of the gyro and accelerometer is the digital pulse signal. It is necessary to convert the pulse signal to an angular increment or velocity increment before it is applied for navigation calculations, and the conversion factor is regarded as the scale factor. The error caused by the inaccuracy of the scale factor is the scale factor error, which is expressed in ppm ($1\times 10^{-6}$). The scale factor error of the high precision RLG is usually $1\times 10^{-6}$ or $1\times 10^{-7}$ orders of magnitude. The scale factor error of gyros ($\Delta {Κ}_{g}$) and scale factor error of accelerometers ($\Delta {Κ}_{f}$) are shown in Equations (3) and (4):

(3) $$\Delta {Κ}_{g}={\left[\begin{array}{cccc} \Delta K_{g_{1}} & \Delta K_{g_{2}} & \cdots & \Delta K_{g_{n}} \end{array}\right]}^{T}$$

($\Delta K_{g_{i}}$ is the scale factor error of the i-th gyro)

(4) $$\Delta {Κ}_{f}={\left[\begin{array}{cccc} \Delta K_{f_{1}} & \Delta K_{f_{2}} & \cdots & \Delta K_{f_{n}} \end{array}\right]}^{T}$$

($\Delta K_{f_{i}}$ is the scale factor error of the i-th accelerometer)

3. Installation Error

**Figure 1:**
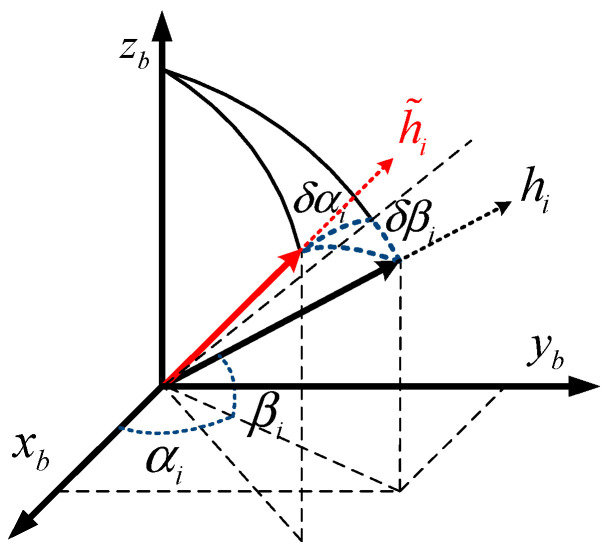
The installation error of the i−th sensor.

Ideally, the direction of the sensitive axis of the gyros and the accelerometers should be in accordance with the system configuration equation. In fact, due to the limitation of the machining and assembly process, there are small angle errors between the input axis of the sensors and the ideal axis. As with the conventional orthogonal IMU, the non-orthogonal RIMU inevitably has installation errors, which are much more complex. In space, there are two error angles between the theoretical installation axis ($h_{i}$) and actual installation axis (${\tilde{h}}_{i}$) of a sensor. The installation error of the i-th sensor is shown in Figure 1.

As is shown in Figure 1, $\beta_{i}$ is the angle between the theoretical installation axis $h_{i}$ and the plane $x_{b}-y_{b}$ of the INS body frame (b). $\alpha_{i}$ is the angle between $x_{b}$ axis and the projection of $h_{i}$ in the plane $x_{b}-y_{b}$. $\delta \alpha_{i}$ and $\delta \beta_{i}$ are the error angles of the actual installation axial and theoretical installation axial of the i-th sensor. 

We use the error of the configuration matrix (ΔH) to describe the installation error. The misalignment error between the actual configuration matrix $\tilde{H}$ and the theoretical configuration matrix H is as follows:

(5) $$\tilde{H}=H+\Delta H=H+\delta \alpha P+\delta \beta Q$$

where $\left\{ \begin{array}{l} \delta \alpha =diag\left[\begin{array}{cccc} \delta \alpha_{1} & \delta \alpha_{2} & \cdots & \delta \alpha_{n} \end{array}\right]\\ \delta \beta =diag\left[\begin{array}{cccc} \delta \beta_{1} & \delta \beta_{2} & \cdots & \delta \beta_{n} \end{array}\right] \end{array}\right.$, 

$P=\left[\begin{array}{ccc} \cos\beta_{1}\sin\alpha_{1} & -\cos\beta_{1}\cos\alpha_{1} & 0\\ \cos\beta_{2}\sin\alpha_{2} & -\cos\beta_{2}\cos\alpha_{2} & 0\\ \vdots & \vdots & \vdots \\ \cos\beta_{n}\sin\alpha_{n} & -\cos\beta_{n}\cos\alpha_{n} & 0 \end{array}\right]$ $Q=\left[\begin{array}{ccc} -\sin\beta_{1}\cos\alpha_{1} & -\sin\beta_{1}\sin\alpha_{1} & \cos\beta_{1}\\ -\sin\beta_{2}\cos\alpha_{2} & -\sin\beta_{2}\sin\alpha_{2} & \cos\beta_{2}\\ \vdots & \vdots & \vdots \\ -\sin\beta_{n}\cos\alpha_{n} & -\sin\beta_{n}\sin\alpha_{n} & \cos\beta_{n} \end{array}\right]$

Obviously, the installation error of the non-orthogonal RINS is more complex than that of the orthogonal system. The error term is increased, and the error angles are no longer small angle errors. 

According to the analysis above and based on the constant bias, the scale factor error, and the installation error, the error model of gyros and accelerometers can be obtained.

(6) $$\delta \omega^{s}={\tilde{\omega }}^{s}-\omega^{s}=\left(diag\left(\Delta K_{g}\right)H_{g}+diag\left(K_{g}\right)G_{g}\right)N_{g_{ib}}^{b}+\varepsilon^{s}$$

(7) $$f^{s}={\tilde{f}}^{s}-f^{s}=\left(diag\left(\Delta K_{f}\right)H_{f}+diag\left(K_{f}\right)G_{f}\right)N_{f_{ib}}^{b}+\nabla^{s}$$

In Equations (6) and (7), $\omega^{s}$ is the angular increment, $f^{s}$ is the velocity increment, $\delta \omega^{s}$ and $\delta f^{s}$ are the error values of $\omega^{s}$ and $f^{s}$, $diag\left(K_{g}\right)$ and $diag\left(K_{f}\right)$ are the diagonal matrices of $K_{g}$ and $K_{f}$, and $N_{g_{ib}}^{b}$ and $N_{f_{ib}}^{b}$ are the pulse outputs of the gyros and accelerometers. The above error equation is the error model of analytical calibration.

## 3. Modeling Method of Systematic Calibration for the Non-orthogonal RIMU

We take the subsystem composed of three gyroscopes and three accelerometers as the research object to study the systematic calibration method of non-orthogonal RIMU. Compared with taking the whole system as the calibration object, using the subsystem as the calibration object has several obvious advantages. Firstly, only the installation error model is different in this method compared with the orthogonal system model. Secondly, due to the small number of devices and the low order of the filter, it is beneficial to improve the calibration accuracy of the system and accelerate the convergence time of error parameters. Finally, the method of using a subsystem as the calibration object can effectively avoid the non-full rank of the configuration matrix and improve the error decoupling efficiency.

In the process of solving the installation error, $N_{g_{ib}}^{b}$ and $N_{f_{ib}}^{b}$ are the outputs of the sensors in the body frame. Then, the output of the gyros and accelerometers ($N_{g_{ib}}^{s}$ and $N_{f_{ib}}^{s}$) must be transformed as follows:

(8) $$N_{g_{ib}}^{b}={\tilde{M}}_{g}N_{g_{ib}}^{s}\;({\tilde{M}}_{g}={\left({\tilde{H}}_{g}{}^{T}{\tilde{H}}_{g}\right)}^{-1}{\tilde{H}}_{g}{}^{T})$$

(9) $$N_{f_{ib}}^{b}={\tilde{M}}_{f}N_{f_{ib}}^{s}\;({\tilde{M}}_{f}={\left({\tilde{H}}_{f}{}^{T}{\tilde{H}}_{f}\right)}^{-1}{\tilde{H}}_{f}{}^{T})$$

From Equations (8) and (9), ${\tilde{M}}_{g}$ and ${\tilde{M}}_{f}$ contain the installation errors; this will inevitably lead to inaccurate calibration results if the model is adopted without modification. In view of the above analysis, we regard all the scale factor errors and installation errors of each device as small values after the analytic calibration. It can be considered that the output of the system is in a pseudo b frame (there are small angle errors in the pseudo b frame ($\tilde{b}$) and the real b frame) after the transformation of the configuration matrix. Therefore, the residual installation errors can be considered as the misalignment angles between the $\tilde{b}$ frame and b frame, while the scale factor errors and constant biases of the sensors are still analyzed with each sensor.

(10) $$M_{g}=\left(I+\Delta G_{g}\right){\tilde{M}}_{g}$$

(11) $$M_{f}=\left(I+\Delta G_{f}\right){\tilde{M}}_{f}$$

The scale factor error of the subsystem can be expressed as follows:

(12) $${\tilde{K}}_{g}=K_{g}+\Delta K_{g}$$

(13) $${\tilde{K}}_{f}=K_{f}+\Delta K_{f}$$

In view of this, the error model can be decomposed into the following forms (taking the calibration model of gyros as an example):

(14) $$\begin{array}{l} \delta \omega^{n}={\tilde{\omega }}^{n}-\omega^{n}=C_{b}^{n}{\tilde{M}}_{g}\left(diag\left({\tilde{K}}_{g}\right)N_{g_{ib}}^{s}\right)+C_{b}^{n}{\tilde{M}}_{g}\varepsilon^{s}\\ -C_{b}^{n}\left(I+\Delta G_{g}\right){\tilde{M}}_{g}\left(diag\left({\tilde{K}}_{g}-\Delta K_{g}\right)N_{g_{ib}}^{s}\right)\\ =\underset{\mathrm{Installation}\;\mathrm{error}\;\mathrm{part}}{\underbrace{-C_{b}^{n}\Delta G_{g}{\tilde{M}}_{g}\left(diag\left({\tilde{K}}_{g}\right)N_{g_{ib}}^{s}\right)}}+\underset{\mathrm{Scale}\;\mathrm{factor}\;\mathrm{error}\;\mathrm{part}}{\underbrace{C_{b}^{n}{\tilde{M}}_{g}\left(diag\left(\Delta K_{g}\right)N_{g_{ib}}^{s}\right)}}+\underset{\begin{array}{l} \mathrm{Constant}\;\\ \mathrm{bais}\;\mathrm{part} \end{array}}{\underbrace{C_{b}^{n}{\tilde{M}}_{g}\varepsilon^{s}}} \end{array}$$

where I is a 3-order identity matrix, $diag\left({\tilde{K}}_{g}\right)$ is the diagonal matrix of ${\tilde{K}}_{g}$, $N_{g_{ib}}^{s}$ is the output of gyros in the s frame, and $M_{g}$ is the installation matrix obtained by the analytic calibration. Moreover, these parameters are all known quantities.

$\Delta G_{g}$ is the residual installation error matrix between the $\tilde{b}$ frame and b frame, $\Delta K_{g}$ is the residual scale factor error of the gyros, and $\varepsilon^{s}$ is the constant bias of gyros in the s frame. These three parts are the error items which need to be calibrated.

The error model can be divided into three parts.

1. The first part is the part of the installation error:

(15) $$\Delta 1=-C_{b}^{n}\left[\begin{array}{ccc} 0 & G_{12} & G_{13}\\ G_{21} & 0 & G_{23}\\ G_{31} & G_{32} & 0 \end{array}\right]{\tilde{M}}_{g}\left(diag\left({\tilde{K}}_{g}\right)N_{g_{ib}}^{s}\right)$$

where $C_{b}^{n}$ can be obtained from alignment results, and ${\tilde{M}}_{g}\left(diag\left({\tilde{K}}_{g}\right)N_{g_{ib}}^{s}\right)$ is the output of gyros in the b frame, which can be modified as $M\left(diag\left(\tilde{K}\right)N^{s}\right)=\omega_{}^{b}={\left[\begin{array}{ccc} \omega_{x}^{b} & \omega_{y}^{b} & \omega_{z}^{b} \end{array}\right]}^{T}$. The installation error includes six items $\left(G_{12},G_{13},G_{21},G_{23},G_{31},G_{32}\right)$. Equation (15) can be simplified as follows:

(16) $$\Delta 1=\left[\begin{array}{cccccc} C_{11}\omega_{y}^{b} & C_{11}\omega_{z}^{b} & C_{12}\omega_{x}^{b} & C_{12}\omega_{z}^{b} & C_{13}\omega_{x}^{b} & C_{13}\omega_{y}^{b}\\ C_{21}\omega_{y}^{b} & C_{21}\omega_{z}^{b} & C_{22}\omega_{x}^{b} & C_{22}\omega_{z}^{b} & C_{23}\omega_{x}^{b} & C_{23}\omega_{y}^{b}\\ C_{31}\omega_{y}^{b} & C_{31}\omega_{z}^{b} & C_{32}\omega_{x}^{b} & C_{32}\omega_{z}^{b} & C_{33}\omega_{x}^{b} & C_{33}\omega_{y}^{b} \end{array}\right]\left[\begin{array}{c} G_{12}\\ G_{13}\\ G_{21}\\ G_{23}\\ G_{31}\\ G_{32} \end{array}\right]$$

2. The second part is the scale factor errors:


(17) $$\Delta 2=C_{b}^{n}{\tilde{M}}_{g}diag\left(\left[\Delta {\tilde{K}}_{g}\right]\right)N_{g_{ib}}^{s}=C_{b}^{n}{\tilde{M}}_{g}diag\left(\left[{\tilde{N}}_{g_{ib}}^{s}\right]\right)\left[\begin{array}{c} \Delta K_{g_{1}}\\ \Delta K_{g_{2}}\\ \Delta K_{g_{3}} \end{array}\right]$$

3. The third part is the constant bias:


(18) $$\Delta 3=C_{b}^{n}{\tilde{M}}_{g}\varepsilon_{}^{s}=C_{b}^{n}{\tilde{M}}_{g}\left[\begin{array}{c} \varepsilon_{1}^{s}\\ \varepsilon_{2}^{s}\\ \varepsilon_{3}^{s} \end{array}\right]$$

The accelerometers have the same error model of gyros.

(19) $$\delta f^{n}=\underset{\mathrm{Installation}\;\mathrm{error}\;\mathrm{part}}{\underbrace{-C_{b}^{n}\Delta G_{a}{\tilde{M}}_{a}\left(diag\left({\tilde{K}}_{a}\right)N_{a_{ib}}^{s}\right)}}+\underset{\mathrm{Scale}\;\mathrm{factor}\;\mathrm{part}}{\underbrace{C_{b}^{n}{\tilde{M}}_{a}\left(diag\left(\Delta K_{a}\right)N_{a_{ib}}^{s}\right)}}+\underset{\begin{array}{l} \mathrm{Constant}\;\\ \mathrm{bais}\;\mathrm{part} \end{array}}{\underbrace{C_{b}^{n}{\tilde{M}}_{a}\nabla^{s}}}$$

In summary, the installation errors of the non-orthogonal RIMU are the same as that of the orthogonal IMU after adopting this calibration model, and the scale factor errors and constant biases of the sensors are determined by the number of the sensors. This greatly simplifies the error model of the non-orthogonal RIMU and makes it possible to separate those errors effectively.

## 4. Experiment and Result Analysis

**Figure 2:**
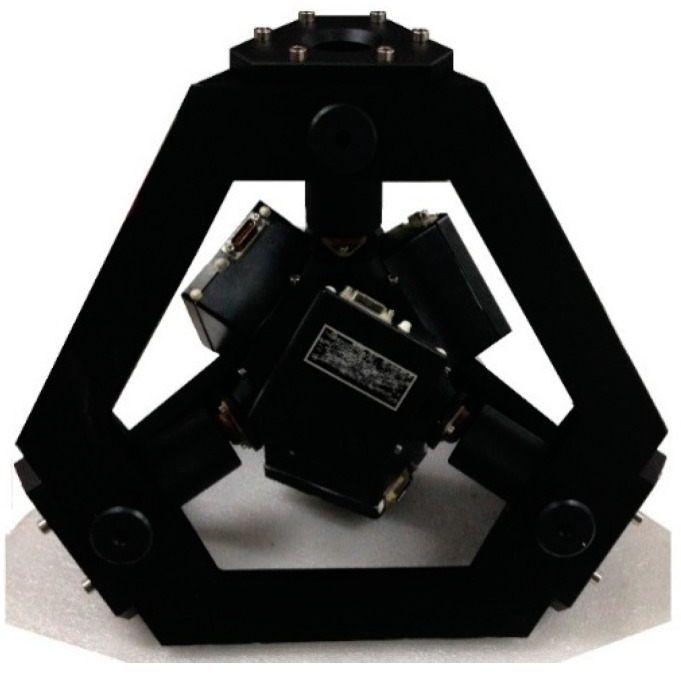
Shape of the RIMU prototype.

As is shown in Figure 2, we designed a prototype consisting of four ring laser gyros and four quartz accelerometers. The bias instability of the gyros used in the prototype is within 0.008 °/h, the random walk of the gyro is within 0.001º/√h, and the bias instability of the accelerometer is within 0.5 mg. 

**Figure 3:**
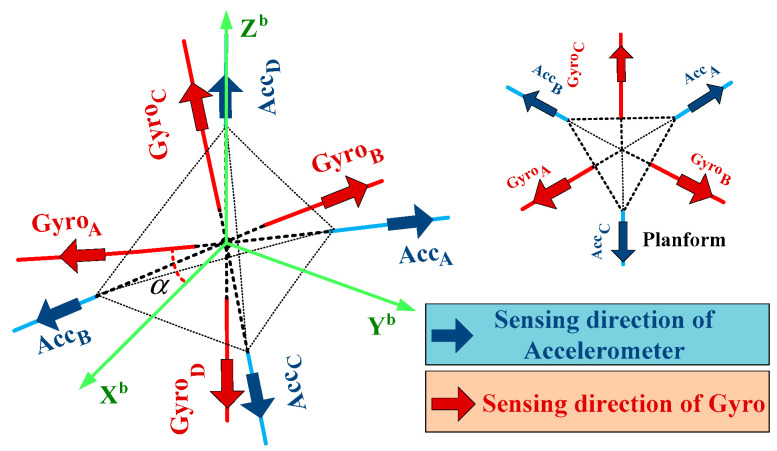
The sensors configuration of the RIMU.

Figure 3 shows the sensors configuration of the prototype. This RIMU adopts the installation method of regular tetrahedron configuration. The sensitive axis of the four gyros is located from the center of the tetrahedron to the center of the four faces, and the sensitive axis of the four accelerometers is directed to the four vertices from the center of the tetrahedron.

**Figure 4:**
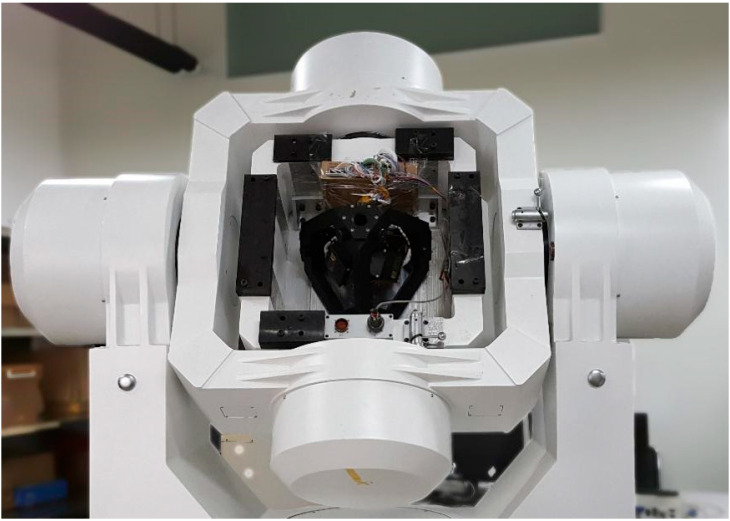
Three-axis turntable for calibration.

The configuration matrix (H) and installation matrix (M) of gyros and accelerometers are as follows:

(20) $$H_{g}=-H_{f}=\left[\begin{array}{ccc} \cos\alpha & 0 & \sin\alpha \\ -\frac{1}{2}\cos\alpha & \frac{\sqrt{3}}{2}\cos\alpha & \sin\alpha \\ -\frac{1}{2}\cos\alpha & -\frac{\sqrt{3}}{2}\cos\alpha & \sin\alpha \\ 0 & 0 & 1 \end{array}\right]$$

(21) $$M_{g}=-M_{f}=\left[\begin{array}{cccc} \frac{2}{3\cos\alpha } & -\frac{1}{3\cos\alpha } & -\frac{1}{3\cos\alpha } & 0\\ 0 & \frac{\sqrt{3}}{3\cos\alpha } & -\frac{\sqrt{3}}{3\cos\alpha } & 0\\ \frac{\sin\alpha }{3\sin^{2}\alpha +1} & \frac{\sin\alpha }{3\sin^{2}\alpha +1} & \frac{\sin\alpha }{3\sin^{2}\alpha +1} & \frac{1}{3\sin^{2}\alpha +1} \end{array}\right]$$

where α=19.4712° is the angle between the A gyro and the $x^{b}$ direction of the IMU. The prototype is installed on the three-axis turntable, which has an attitude accuracy within 1″, as shown by Figure 4.

Using the analytical calibration modeling method in Section 2, the calibration results of each sensor can be obtained. Table 1 shows the gyros’ calibration results of analytical calibration. Table 2 shows the accelerometers’ calibration results of analytical calibration.

After the analytical calibration is completed, the Kalman filtering systematic calibration method is used to complete further estimation of the residual error of the parameters. Based on the error model described in Section 3, the state vector of the subsystem is defined as:

(22) $$X={\left[\begin{array}{ccccc} \varphi^{T} & \delta v^{T} & \delta P^{T} & X_{Gyro}^{T} & X_{Acc}^{T} \end{array}\right]}^{T}$$

(23) $$X_{Gyro}^{}={\left[\begin{array}{ccccccccc} G_{g_{21}}^{} & G_{g_{31}}^{} & G_{g_{32}}^{} & \Delta K_{g_{1}}^{} & \Delta K_{g_{2}}^{} & \Delta K_{g_{3}}^{} & \varepsilon_{1}^{} & \varepsilon_{2}^{} & \varepsilon_{3}^{} \end{array}\right]}^{T}$$

(24) $$\begin{array}{l} X_{Acc}^{}=[\begin{array}{ccccccc} G_{a_{12}} & G_{a_{13}} & G_{a_{21}} & G_{a_{23}} & G_{a_{31}} & G_{a_{32}} & \end{array}\\ {\begin{array}{cccccc} \Delta K_{a_{1}} & \Delta K_{a_{2}} & \Delta K_{a_{3}} & \nabla_{1} & \nabla_{2} & \nabla_{3} \end{array}]}^{T} \end{array}$$

Variables in Equation (22) include three errors of attitude angle ($\varphi =\left[\begin{array}{ccc} \phi_{Pitch} & \phi_{Roll} & \phi_{Heading} \end{array}\right]$, where $\phi_{Pitch}$, $\phi_{Roll}$, and $\phi_{Heading}$ are pitch, roll, and heading errors of the RIMU), three errors of velocity ($\delta v=\left[\begin{array}{ccc} \delta v_{E} & \delta v_{N} & \delta v_{U} \end{array}\right]$, where $\delta v_{E}$, $\delta v_{N}$, and $\delta v_{U}$ are eastern, northern, and vertical velocity errors of the RIMU), three errors of position ($\delta P=\left[\begin{array}{ccc} \delta \lambda & \delta L & \delta h \end{array}\right]$, where δλ, δL, and δh are longitude, latitude, and vertical position errors of the RIMU), calibration parameter errors of gyros ($X_{Gyro}$), and calibration parameter errors of accelerometers ($X_{Acc}$) [19,20,21,22]. 

The errors in the state vector are estimated by the Kalman filter. For the four redundant inertial navigation system, the parameters of all sensors can be obtained by selecting two subsystems for calibration.

**Figure 5:**
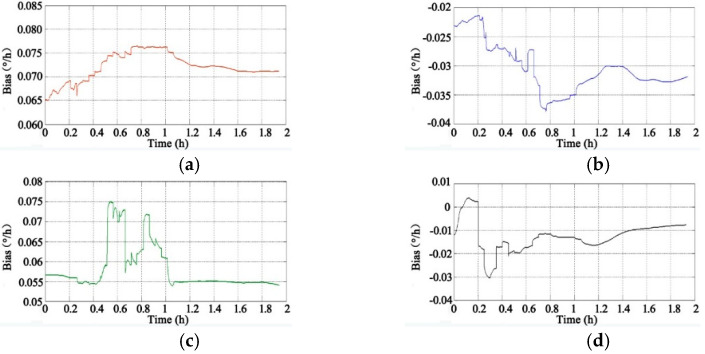
Convergence process of gyros’ constant biases: (**a**) Constant Bias of Gyro A; **(b**) Constant Bias of Gyro B; (**c**) Constant Bias of Gyro C; (**d**) Constant Bias of Gyro D.

**Figure 6:**
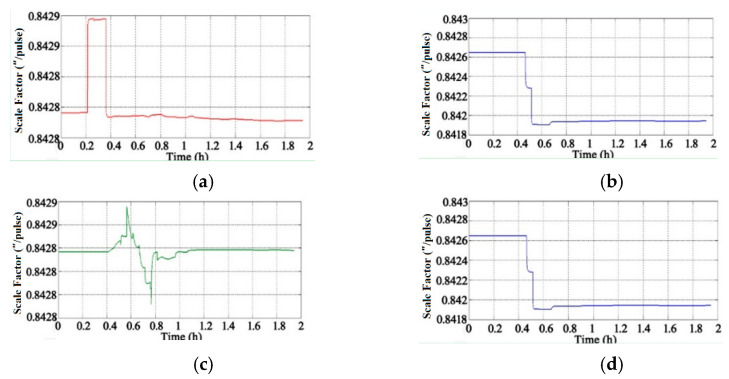
Convergence process of gyros’ scale factors: (**a**) Scale Factor of Gyro A; (**b**) Scale Factor of Gyro B; (**c**) Scale Factor of Gyro C; (**d**) Scale Factor of Gyro D.

**Figure 7:**
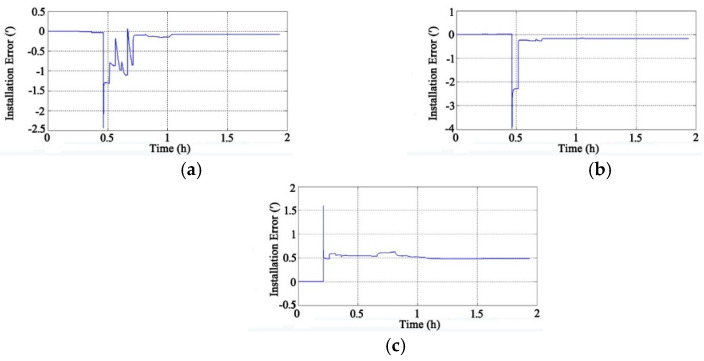
Convergence process of gyros’ installation errors: (**a**) Y-Z Installation Error of Gyros; (**b**) Z-X Installation Error of Gyros; (**c**) Z-Y Installation Error of Gyros.

**Figure 8:**
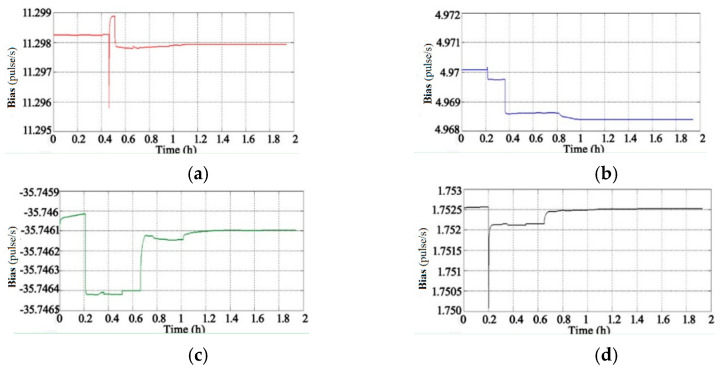
Convergence process of accelerometers’ constant biases: (**a**) Constant Bias of Accelerometer A; (**b**) Constant Bias of Accelerometer B; (**c**) Constant Bias of Accelerometer C; (**d**) Constant Bias of Accelerometer D.

**Figure 9:**
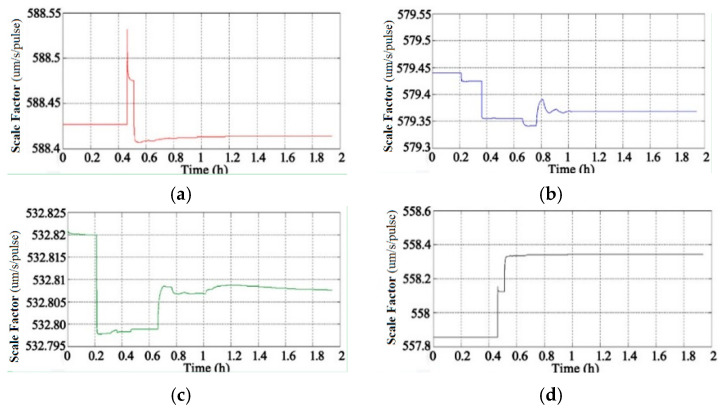
Convergence process of accelerometers’ scale factors: (**a**) Scale Factor of Accelerometer A; (**b**) Scale Factor of Accelerometer B; (**c**) Scale Factor of Accelerometer C; (**d**) Scale Factor of Accelerometer D.

**Figure 10:**
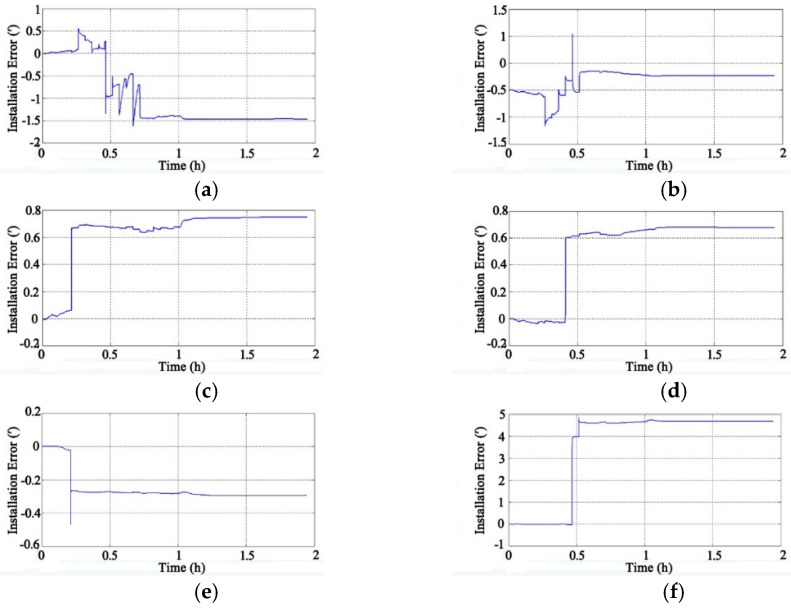
Convergence process of accelerometers’ installation errors: (**a**) X-Y Installation Error of Accelerometers; (**b**) X-Z Installation Error of Accelerometers; (**c**) Y-X Installation Error of Accelerometers; (**d**) Y-Z Installation Error of Accelerometers; (**e**) Z-X Installation Error of Accelerometers; (**f**) Z-Y Installation Error of Accelerometers.

Through the adoption of the rotational excitation designed by Camberlein, calibrated errors can be represented by the rate error and its variation [12]. In the filtering process, the residual errors of calibration are estimated and compensated in real-time, and the calibration results of Equation (16) to Equation (18) can be obtained. The convergence process of the calibration parameters is shown in Figure 5, Figure 6, Figure 7, Figure 8, Figure 9 and Figure 10.

Figure 5 shows the convergence process of the constant biases (the unit is degrees per hour, °/h) of the four gyros. According to the rotation layout scheme, there are 21 positions in a cycle, and the time is about 1 h. The constant biases of the gyros obtained by analytical calibration are used as the initial value. It can be seen that the convergence speed of the gyros’ biases is relatively slow: the results tend to converge after 1.6 h.

Figure 6 shows the convergence process of the gyros’ scale factors (the unit is arc seconds per pulse, ″/pulse). The scale factors of the gyros obtained by analytical calibration are used as the initial value, and the results tend to converge after 1.2 h. 

Figure 7 shows the convergence process of the installation errors (the unit is arc minutes, ′) of the four gyros. The initial value of the installation error is zero, and the results tend to converge after 1.2 h.

Figure 8 shows that the convergence process of the accelerometers’ constant biases (the unit is pulse per second, pulse/s) is obviously faster than that of the gyros’ constant biases: the results tend to converge after 1.2 h. 

Figure 9 shows the convergence process of the accelerometers’ scale factor (the unit is micron per second per pulse, um/s/pulse); the results tend to converge after 1.2 h.

Figure 10 shows the convergence process of the accelerometers’ installation errors (the unit is arc minutes, ′); the results tend to converge after 1.2 h. From Figure 5 to Figure 10, it can be seen that parameters of the gyros and accelerometers have high observability and each error reaches convergence within 2 h. The scale factor and installation error of the devices reach convergence within 1.2 h after calibration, but zero bias of the gyros takes at least 1.6 h to reach convergence. The systematic calibration results are shown in Table 3 and Table 4.

**Figure 11:**
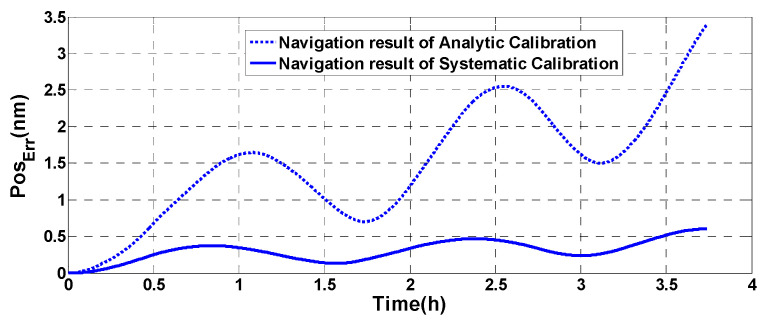
Navigation result of the two calibration parameters.

Two groups of calibration parameters are used to conduct navigation tests in order to compare the calibration accuracy of traditional analytical calibration with the systematic calibration presented in this paper. We use the RIMU outputs in the rotation process of systematic calibration as the rotational spectrum. Because there is only rotation but no line motion in the experiment, the position and velocity are not changed. Therefore, the navigation results can be used to measure the accuracy of the two sets of calibration parameters. The navigation position error of the two calibration parameters is shown in Figure 11.

From Figure 11, after 3.5 h of navigation, the maximum position error of the analytic calibration parameters is 2.5 nm, but the maximum position error of systematic calibration parameters is only 0.5 nm. The results show the superiority of the systematic calibration method, and the validity of the error modeling method is verified at the same time.

**Figure 12:**
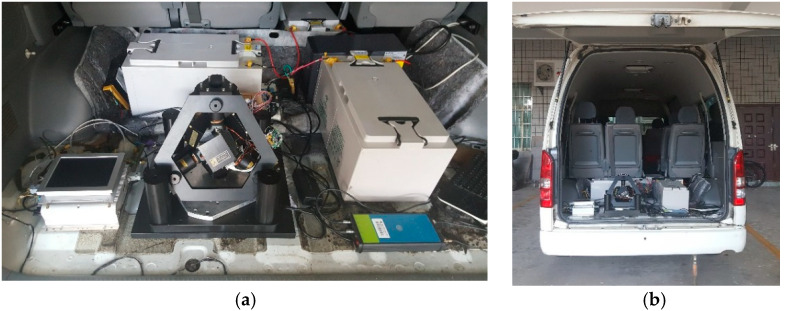
Laboratory equipment photos of dynamic vehicle test: (**a**) photograph of the equipment installation; (**b**) photograph of the test vehicle.

On the basis of laboratory static tests, we also carried out a dynamic vehicle test, located in Hunan, Changsha Province. The experimental equipment included the test car, batteries, uninterrupted power supply (UPS), GPS (the real time positioning precision of the GPS receiver was about 10 m), navigation computer, and RINS. Figure 12a shows the photograph of the equipment installation, while Figure 12b shows the photograph of the test vehicle.

**Figure 13:**
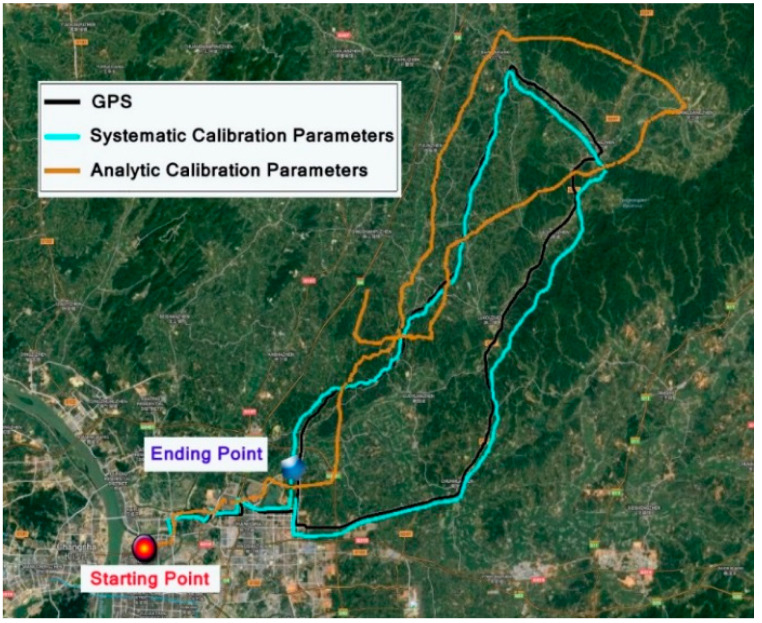
Experimental route of dynamic vehicle test.

The experiment lasted for 3.5 h and the vehicle test distance was close to 150 km. The route of the vehicle is shown in Figure 13. The black line in the figure is the positioning result of the GPS, while the blue line is the navigation route of the RINS using systematic calibration parameters. 

**Figure 14:**
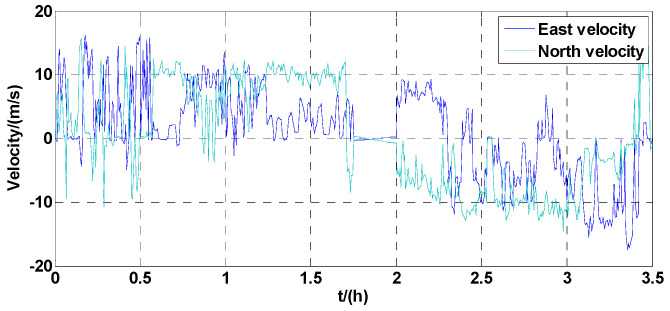
The RINS velocity output in the dynamic vehicle test.

The car started from the starting point (28.2306112.9957) and went eastward, then traveled clockwise to reach the ending point (28.2805, 113.1186). The positioning results of the GPS and navigation results of the RINS using analytic calibration parameters and systematic calibration parameters are shown in the figure, respectively. The RINS velocity output using systematic calibration parameters is shown in Figure 14.

**Figure 15:**
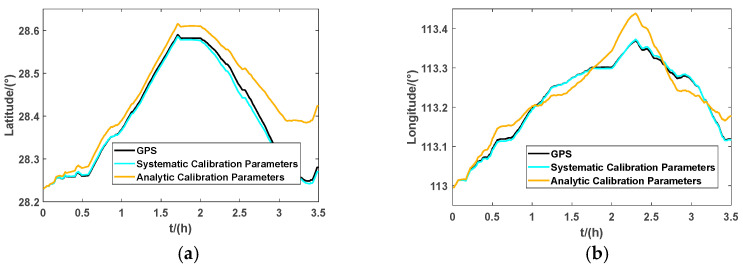
Latitude and longitude output of GPS and RINS: (**a**) latitude output of GPS and RINS; (**b**) longitude output of GPS and RINS.

From Figure 14, apart from the 15 min parking period between 1.75 h to 2 h, the vehicle had been in motion during the entire experiment. While the vehicle was running, the maximum of the east velocity and north velocity was about 15 m/s, and the average velocity was about 10 m/s. To ensure the diversity of the vehicle movement process, there were acceleration and deceleration processes and a large attitude rotation process. This dynamic experiment can effectively test the dynamic performance of IMUs, and then verify whether the calibration parameters are valid. The longitude and latitude outputs of the GPS and RINS are shown in Figure 15. (Based on data generated from the GPS, the parameters of analytic calibration and systematic calibration are compared with each other.)

**Figure 16:**
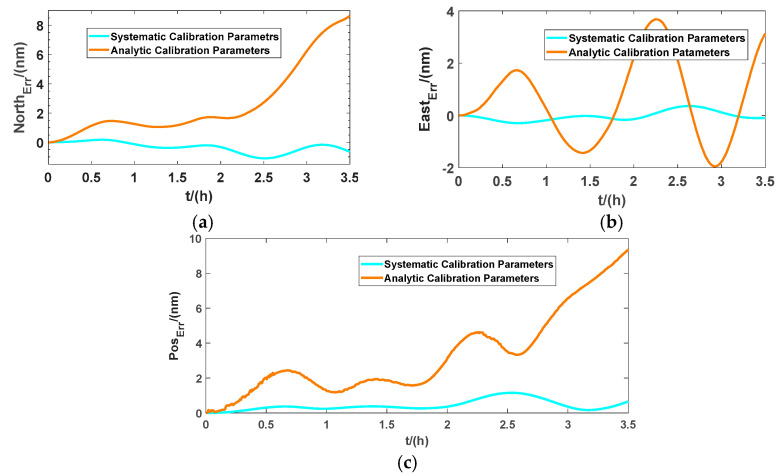
Position errors of RINS: (**a**) north position error; (**b**) east position error; (**c**) overall position error of RINS.

Figure 15 shows an intuitive graphic of the longitude and latitude outputs of the GPS and RINS using analytic calibration parameters and systematic calibration parameters. It can be seen that the positioning output using systematic calibration parameters of the RINS can track the position of the GPS very well, but the navigation results based on analytic calibration are not as good as those of systematic calibration. In order to display the position errors of INS more intuitively, we used the nautical mile (nm) to measure the navigation errors of the system, which are shown in Figure 16.

From Figure 16, the maximum error of the north position is within 1.1 nm, and the maximum error of the east position is within 0.4 nm. The overall position errors reach their maximum value at 2.5 h of navigation, with a maximum value of less than 1.2 nm. On the basis of considering the accuracy limitation of the gyros and accelerometers adopted in the prototype and the residual errors of alignment, it can be concluded that the calibration method in this paper has been used to estimate the related parameters of INS accurately, and the calibration parameters can effectively guarantee the dynamic accuracy of the RINS.

## 5. Conclusions and Future Work

A new modeling method of systematic calibration for the non-orthogonal RINS is presented in this paper. The error model of the non-orthogonal RINS was introduced, and the installation errors caused by the non-orthogonal installation of the sensors were analyzed in detail. Based on the condition that the installation errors of the sensors are small angle errors after the analytic calibration, the outputs of the sensors are transformed into a pseudo body frame. Based on this idea, the error model of the non-orthogonal RIMU is greatly simplified, which makes it possible to achieve the high accuracy parameters calibration. This modeling method has a strong universality, and is suitable for various systems with different redundancy and configuration schemes.

On the basis of theoretical analysis, the accuracy of the calibration model was studied by laboratory static test and dynamic vehicle test. A prototype consisting of four ring laser gyros and four quartz accelerometers was taken as the experimental object. In the static navigation experiment, the accuracy of the navigation results obtained by the calibration model in this paper was obviously higher than that using the traditional analytic calibration method. In the dynamic vehicle test, the maximum error of the RINS was within 1.2 nm in 3.5 h under large maneuvering conditions. It is considered that the calibration parameters can effectively guarantee the dynamic performance of the system, and then the validity of the modeling method was also proved.

At present, the rotational excitation method is also using the traditional orthogonal system excitation method. In the future work, the observability of the filter correlation errors will be analyzed to find a more effective error excitation method for the non-orthogonal redundant inertial navigation system.

## Figures and Tables

**Table 1:** Gyros’ calibration results of analytical calibration.

Constant Bias (deg/h)	Scale Factor (arcsec/P)	Installation Error (Arcmin)		
0.0653212	0.8428342	−0.50137276	12.40504038	1.41809631
−0.0238744	0.8426311	−7.30134007	2.41414393	−16.239074
0.0567933	0.8428741	3.43666491	1.59163083	8.75886152
−0.0120432	0.8428291	5.00401933	0	0
	$H_{g}$	0.94252310	0.00225294	0.3341420
		−0.47225387	0.81747758	0.32972919
		−0.4710470	−0.81561072	0. 3600881
		0.00066746	0	−1
$M_{g}$	0.7068790	−0.3543804	−0.353238	0.00065704
	0.0022580	0.6138032	−0.610860	−0.0021112
	0.2504770	0.2491095	0.2503716	−0.7500393

**Table 2:** Accelerometers’ calibration results of analytical calibration.

Constant Bias (P/s)	Scale Factor (um/s/P)	Installation Error (Arcmin)		
11.2998213	588.42632	−0.98297969	7.74504177	2.78028643
4.970311	579.44373	−2.91986040	3.37245968	−12.39011157
−35.746083	532.82109	1.22897098	3.04536398	9.19761525
1.752673	557.84876	2.29457806	0	0
	$H_{f}$	0.94266319	0.00360847	0.33374584
		−0.47352839	0.81719882	0.32860957
		−0.47040483	−0.81603359	0.33588118
		0.00145561	0	−1
$M_{f}$	0.70666156	−0.3549895	−0.352371	0.0008374
	0.00336339	0.61360337	−0.610945	−0.0024465
	0.2507754	0.248550	0.2500148	−0.7506532

**Table 3:** Gyros’ calibration results of systematic calibration.

Constant Bias (deg/h)	Scale Factor (arcsec/P)	Installation Error (Arcmin)	
0.07114045	0.8428114	$G_{g_{21}}$	−0.07325445
−0.03186325	0.8419425	$G_{g_{31}}$	−0.1645371
0.05400473	0.8428379	$G_{g_{32}}$	−0.4983532
−0.007630583	0.8437471		

**Table 4:** Accelerometers’ calibration results of systematic calibration.

Constant Bias (P/s)	Scale Factor (um/s/P)	Installation Error (Arcmin)			
11.29792	588.4141	$G_{f_{12}}$	−1.493214	$G_{f_{13}}$	0.2543218
4.968388	579.3682	$G_{f_{21}}$	0.7457321	$G_{f_{23}}$	0.6882742
−35.74609	532.8076	$G_{f_{31}}$	−0.2893257	$G_{f_{32}}$	4.713258
1.752524	558.3433				

## Data Availability

Not applicable.
